# The Investigation of High-Temperature SAW Oxygen Sensor Based on ZnO Films

**DOI:** 10.3390/ma12081235

**Published:** 2019-04-15

**Authors:** Lin Shu, Xuemin Wang, Dawei Yan, Long Fan, Weidong Wu

**Affiliations:** 1 Science and Technology on Plasma Physics Laboratory, Research Center of Laser Fusion, China Academy of Engineering Physics, Mianyang 621900, Sichuan, China; wangxuemin75@sina.com (X.W.); dawei.yan@hotmail.com (D.Y.); sfanlong@163.com (L.F.); 2 Collaborative Innovation Center of IFSA (CICIFSA), Shanghai Jiao Tong University, Shanghai 200240, China

**Keywords:** surface acoustic wave, ZnO, oxygen gas sensor, high temperature

## Abstract

In this paper, a wireless oxygen sensor based on a surface acoustic wave (SAW) was reported. For high-temperature applications, novel Al_2_O_3_/ZnO/Pt multilayered conductive film was deposited on langasite substrate as the electrodes, and ZnO film obtained by the pulse laser deposition (PLD) method was used as the sensitive film. The measurements of X-ray diffraction (XRD) and a scanning electron microscope (SEM) showed that the c-axis orientation of the ZnO grains and the surface morphology of the films were regulated by the deposition temperature. Meanwhile, the gas response of the sensor was strongly dependent on the surface morphology of the ZnO film. The experimental results showed that the oxygen gas sensor could operate at a high-temperature environment up to 850 °C with good stability for a long period. The max frequency shift of the sensors reaches 310 kHz, when exposed to 40% O_2_ gas at 850 °C. The calculated standard error of the sensors in a high-temperature measurement process is within 3%. Additionally, no significant signal degradation could be observed in the long-term experimental period. The prepared SAW oxygen gas sensor has potential applications in high-temperature sensing systems.

## 1. Introduction

There have been growing demands for oxygen gas sensors in a wide range of applications, such as the environment protection, food security, automotive, chemical industry and artificial intelligence fields [1,2,3,4,5]. In particular, high temperature gas sensors are required in engines and combustions systems for the detection of combustion efficiency to reduce pollution [6]. In these applications, the operation temperature reaches 650 °C or even higher. Very few technological options are available for sensing in a high-temperature environment. There are two core problems in high temperature-sensing applications. On the one hand, the devices are apt to become invalid when exposed to overheating. The decomposition and degradation of the materials used to fabricate the sensors would result in the irreversible destruction of sensors [7]. On the other hand, the sensors are hard to power electrically for a long time by a wired method when operate at high temperature [8]. Among the variety of oxygen gas sensors, such as electrochemical sensors, semiconducting sensors, fiber optic sensors and quartz crystal microbalance (QCM) sensors, the surface acoustic wave (SAW) gas sensor represents an attractive solution for harsh-environment applications for its capacities of high-temperature operation, and wireless and passive detection [9,10]. 

A typical SAW gas sensor is composed of a SAW resonator and a sensitive layer. Owing to chemical and physical absorption of gas species on the sensitive layer, the sensor’s surface charge transfer and associated mass increase during a reaction with target gas molecules [11]. Because the acoustic wave energy is mainly concentrated on the surface of the SAW sensors, any perturbations in in mass density and electrical conductivity on the surface of the SAW sensors will make a significant impact on the acoustic propagation [12]. Therefore, SAW gas sensors have the properties of high sensitivity, fast response and high accuracy [13]. The key factor in the design of a SAW gas sensor is the sensitive layer. In recent years, numerous materials including semiconducting metal oxides (TiO_2_, SnO_2_, ZnO, Pt-ZnO, Fe-ZnO), metal-organic framework materials and polymers, have been applied in oxygen gas sensors due to their high sensitivity and rapid response [14,15,16,17]. However, due to the poor stability and low sensitivity of the sensitive film [18], oxygen gas sensors for the high-temperature applications (>800 °C) were rarely reported. 

In previous works, SAW sensors based on langasite (LGS, La_3_Ga_5_SiO_14_) substrate and multilayer laminated film electrodes for high temperature applications have been investigated [19,20]. Following this research, ZnO thin film was deposited on the sensor to form a high-temperature oxygen gas sensor in this work. The performance of the realized SAW sensors was characterized up to 800 °C with O_2_ concentration from 1% to 40%. Meanwhile, the dependence of ZnO sensitive films with different grain size and surface morphology on their sensing performance was studied and discussed. The experimental results indicated that the SAW sensor realized had high potential use in harsh environmental sensing applications.

## 2. Experimental Setup

**Figure 1:**
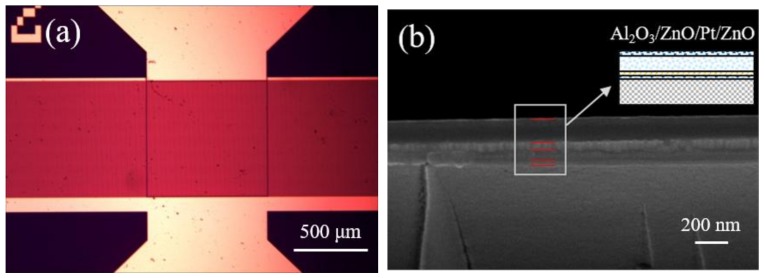
(**a**) Photo of surface acoustic wave (SAW) resonators, (**b**) the sectional view scanning electron micrograph (SEM) of the multilayer laminated electrodes.

In this work, a SAW resonator with operating frequency of 335 MHz was simulated by using the finite element method (FEM) firstly. A one-port SAW resonator, which consists of an interdigital transducer (IDT) and two reflector banks, was fabricated on the LGS substrates by photolithography and etching techniques. The IDT had 100.5 pairs of electrodes with a width of 2 μm and finger spacing of 2 μm. The reflector banks contained 250 short-circuited gratings at both sides of the IDT. The acoustic aperture is 800 μm. The photos of the SAW resonator are shown in Figure 1a. For high-temperature applications, the multilayer laminated conducting electrodes, including a 10-nm-thick Al_2_O_3_ film as an oxygen-ion diffusion barrier layer, a 30-nm-thick ZnO film as buffer layer, and a 70-nm-thick Pt film as conducting layer were prepared. The sectional view scanning electron microscopy (SEM) of the electrodes is shown in Figure 1b. The growth method of the multi-layered conducting film was reported in detail in the previous work. 

The ZnO sensitive films on the top of the whole SAW resonator were prepared by the PLD (pulse laser deposition) method. By adjusting the deposition conditions, the crystallizing quality of the ZnO films were regulated and the thickness of ZnO films (*t*_ZnO_) could be controlled. Before measurement, the sensors were annealed to improve their thermal stability. 

The crystal structures of the ZnO sensitive films were characterized by X-ray diffraction (XRD, Cu-Kα, Bede-D1, Bede Co., UK). The c-axis orientation degree of the ZnO films were characterized by the full width at half maximum (FWHM) of ZnO diffraction peak. The *t*_ZnO_ of the samples was measured by a profilometer (Dektak 150, Veeco Co., New York, USA). Meanwhile, the surface morphology of the ZnO films was observed by SEM (JSM-6460, JEOL Ltd., Japan).

**Figure 2:**
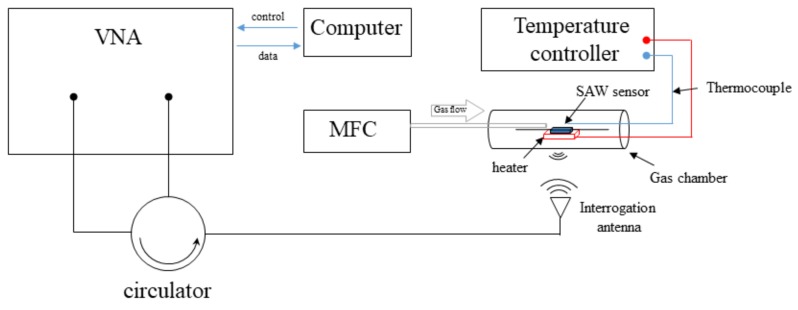
The schematic diagram of the experimental setup.

For wireless detection, the SAW sensors were attached to a λ/4 dipole antenna which consists of two 15-cm-length conductor wires made of 0.5 mm diameter copper wire. The schematic diagram of the experimental setup, mainly containing a mass flow controller (MFC) unit (E-lite, Beijing), a vector network analyzer (VNA, Agilent E5071b, Agilent, USA), and a temperature-controlled gas cell, was shown in Figure 2. The prepared SAW gas sensors were continuously measured with oxygen concentration up to 40%. The operating temperature was from 650 °C to 850 °C. The measurements of the reflection-scattering parameters (S_11_) were acquired and processed in a computer by homemade software. At last, the stability of the sensors in a high-temperature environment was verified in a period of 100 h.

## 3. Results and Discussions

### 3.1. Structural Characterization

**Figure 3:**
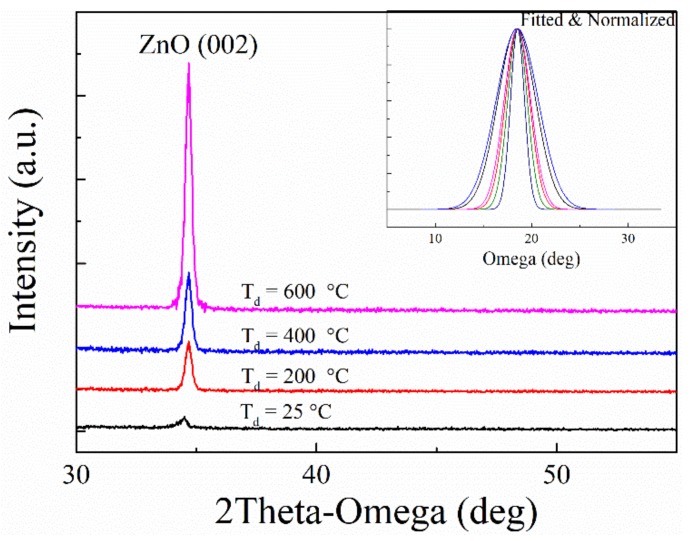
X-ray diffraction (XRD) pattern of ZnO thin films deposited at different temperature.

Due to its great influence on the grain growth, the deposition rate modulated by deposition temperature (*T*_d_) is regarded as one of the key factors in film deposition [21]. ZnO films with different *T*_d_ emerged with different crystallinity and surface morphology. The XRD θ-2θ patterns of the ZnO film deposited on LGS substrate were presented in Figure 3. In the case of ZnO pristine films, the patterns showed reflection peaks at 34.68°, corresponding to ZnO (002) reflections. The pattern exhibited good agreement with the standards card (JCPDS, Joint Committee on Powder Diffraction Standards). With the increase of *T*_d_of ZnO films, the intensity of diffraction peaks of ZnO increased gradually, indicating the better c-axis orientation of ZnO film. The FWHM (full width at half maximum) of ZnO (002) diffraction peak was calculated and it decreased from 10.2 to 2.6 when *T*_d_ increased from 25 °C to 600 °C. The parameters of the samples prepared in this work are presented in Table 1.

**Figure 4:**
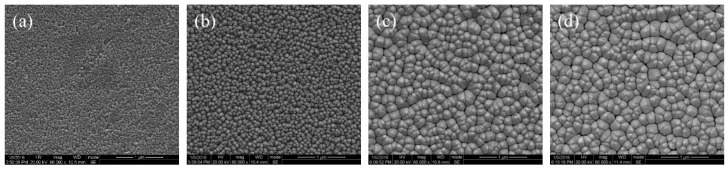
Top-view SEM micrograph of ZnO films deposited at different conditions, (**a**) sample I, (**b**) sample II, (**c**) sample III, (**d**) sample IV.

**Figure 5:**
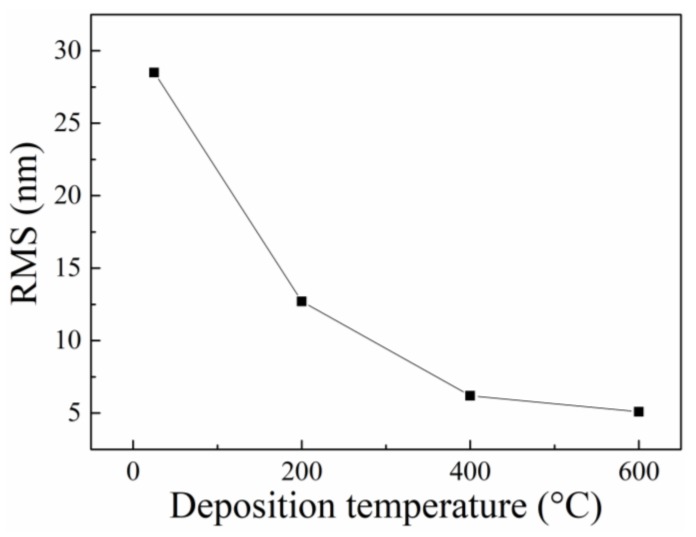
The dependence of root mean square roughness (RMS) of ZnO film on different deposition temperature.

The top-viewed SEM characterizations of the ZnO films are presented in Figure 4, showing good uniform surface for the ZnO films. With low deposition temperature (*T*_d_ = 25 °C and *T*_d_ = 200 °C), the ZnO grain detached from each other and formed as grain islands, as shown in Figure 4a,b. From the statistics of the two samples, the average ZnO grain size was about 42 nm and 51 nm, respectively. When enhancing *T*_d_ to 400 °C and 600 °C, the size of ZnO grain grew and there existed almost no pores, as shown in Figure 4c,d. The ZnO grain also grew and the average grain size reached about 141 nm with *T*_d_ = 600 °C. Meanwhile, the surface roughness of the films also varied with different *T*_d_. the RMS (root mean square roughness) of the films were extracted and its correspondence to *T*_d_ were presented in Figure 5. It could be found that the RMS of the samples increased significantly with decreasing of *T*_d_, indicating the ZnO film with low *T*_d_ had a larger surface roughness than the samples with higher *T*_d_. The mechanism of the surface morphology variation in the thin film deposition process was demonstrated by Bartelt et al. [22] and Xia et al. [23]. Generally, the grain island size was dependent on the grain diffusion rate and the deposition rate, which were determined by the deposition temperature. In this work, the grain size of ZnO film deposited at lower *T*_d_ was small, but its surface morphology was coarsest among the samples. This evidence demonstrated that the ZnO film deposited at low temperature has a bigger specific surface area than the others, indicating that the film could adsorb more target gas molecules when exposed to gas surroundings.

### 3.2. High Temperature Operation Property and Discussion

**Figure 6:**
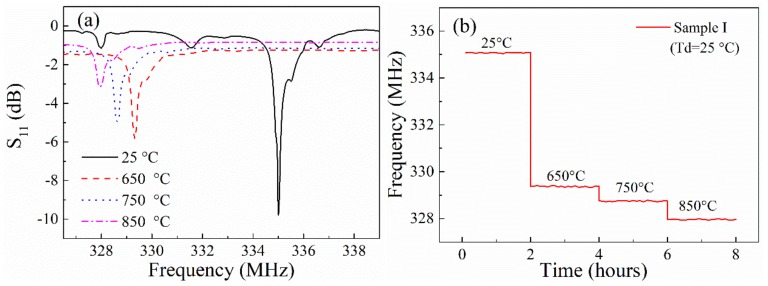
(**a**) the S_11_ parameters of the SAW sensor (sample I) at operation temperature of 25 °C, 650 °C, 750 °C and 850 °C; (**b**) long-term measurements of the SAW sensor at different temperature.

Firstly, the sensors were tested at 25 °C, 650 °C, 750 °C, and 850 °C. An example of one sample’s response to the variation of temperature is shown in Figure 6a. It can be seen that the response of the sensor exhibited a negative shift in the resonance frequency when heated up, showing good agreement with the LGS SAW sensors in the previous work. On one hand, the wavelength *λ* of the SAW device increases with increasing temperature owing to thermal expansion effects. On the other hand, the SAW velocity decreases with increasing temperature because of its negative temperature coefficient of velocity of the LGS substrate. both of the two factors have negative effects on the resonance frequency *f*_r_, where *f*_r_ = *v*_s_/*λ*. Furthermore, we find that the resonant intensity of the SAW sensor decreased with the operation temperature increasing from 650 °C to 850 °C. This is mainly caused by the variation in resistance of the copper wire used as the dipole antenna, leading to the resistance mismatch between the SAW sensor and antenna. However, the SAW sensor retains good performance at 850 °C, as its calculated quality factor reached 658. Additionally, the SAW sensor exhibits good stability at high temperature, as no significant frequency drift of the sensor could be observed in the long-term heating measurement, as shown in Figure 6b. The maximum deviation rate of the resonance frequency at each temperature point is much less than 0.1%. 

### 3.3. O_2_ Gas-Sensing Performance

**Figure 7:**
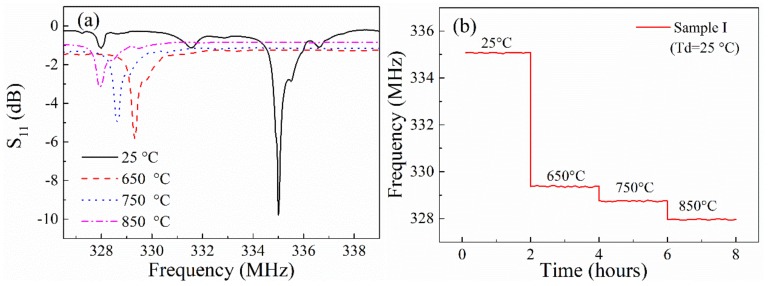
The response properties of the sensors with different concentration of O_2_ at 850 °C. (**a**) response of sensors versus time and (**b**) response of the sensors versus O_2_ concentration.

Figure 7 shows the dynamic response of the four SAW sensors covered with different ZnO films to O_2_ gas. The concentration of O_2_ varies from 1% to 40%, and the operation temperature is 850 °C. The response to the target gas is defined as the frequency shift (Δf). From Figure 7, the exposure of the sensors to O_2_ resulted in a rapid decrease of *f*_r_and the sensors reached a stable saturation response in a few seconds. When the N_2_ gas blew in, the frequency of the sensors returned to the baseline. No significant frequency shift in the baseline of the sensors is observed in this work, demonstrating good repeatability of the sensors. Obviously, sample I with the low *T*_d_ has higher sensing sensitivity to the target gas than the other three samples, indicating that the sensing performance is affected by the surface morphology of the ZnO films. The dependence of resonance frequency shift on O_2_ concentrations was plotted in Figure 7b. It can be observed that the frequency shift of the sensors increases with the increasing O_2_ concentration. However, the sample III and sample IV had a smaller frequency shift at the same conditions than the others. Among the four sensors, sample I has the strongest sensitivity to O_2_ gas. It shows a frequency shift of 315.1 kHz to O_2_ gas at a concentration of 40%, which is about 20 times stronger than that of sample IV. The enhancement is attributed to the defective surface morphology of sample I. The large specific surface area was obtained owing to the coarse surface of the ZnO films, resulting in the improvement of the capability in the absorbing target gas molecule and contributing to the gas response of the sensors [24]. Moreover, large amount of donor defects, such as oxygen vacancy (V_o_) and zinc interstitial (Zn_i_), was introduced by the rough surface of the ZnO film. They provided electrons for the absorbed oxygen molecule and enhanced the probability of interaction between the ZnO film and O_2_ gas molecules, subsequently [25]. Therefore, the higher response of the sensors exhibited in the samples with lower ZnO deposition temperature.

**Figure 8:**
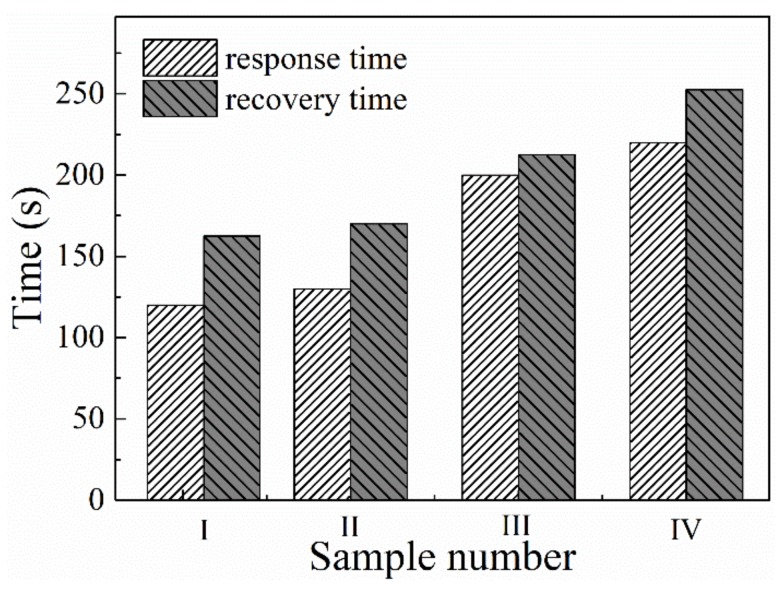
The response and recovery time of the sensors at 850 °C.

Figure 8 shows the response and recovery characterizations of the samples. The response and recovery time is defined as the time needed to reach the 90% of the maximum response or the initial baseline. As shown in the figure, sample I has the shortest response and recovery time, about 122s and 165s, while the response and recovery time of sample IV reached 219s and 251s, respectively. It can be concluded that both of the response and recovery time increased when the *T*_d_ increased. In addition, It should be noted that both of the response and recovery time of the sensors show little change when operated at different O_2_ concentrations. 

**Figure 9:**
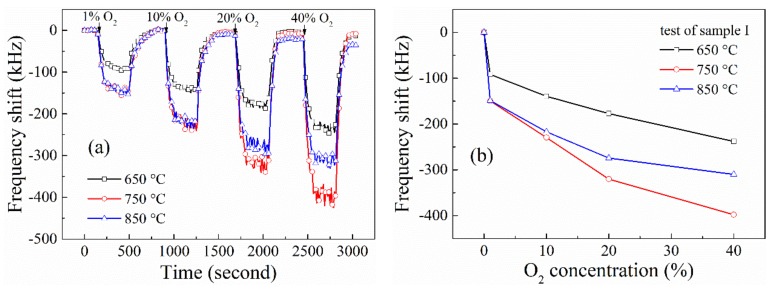
The response properties of sample I at different temperature with O_2_ concentration increases to 40%. (**a**) response of sensors versus time and (**b**) response of the sensors versus O_2_ concentration.

**Figure 10:**
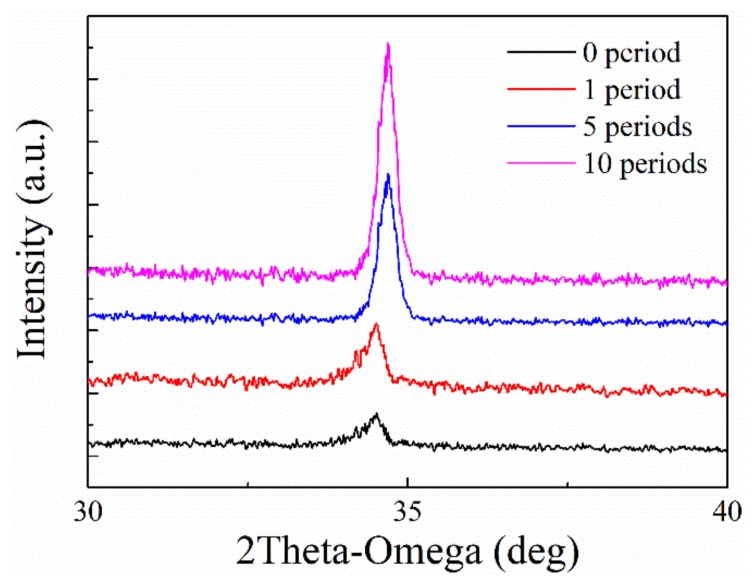
XRD pattern of ZnO thin films (sample I) characterized after 0, 1, 5 and 10 high-temperature experimental periods.

Moreover, the sensors had different response to O_2_ at different temperature. Taking sample I for example, the dependency of its response to O_2_ concentration at different temperature was presented in Figure 9. Obviously, the sensitivity of the sensor increases as the operation temperature increases from 650 °C to 750 °C. However, there exists a negative influence to the sensitivity of the sensors when heated up to 850 °C. When the concentration of the O_2_ gas reaches 20%, the sensor exhibits a stronger response at 750 °C than that at 850 °C. This phenomenon is also observed in the measurements of the other three samples, which conflicts with similar research operated at low temperature. We speculate this is caused by the modification of the ZnO films under the specific condition. When operated at high-temperature and oxidizer rich surroundings, the ZnO-sensitive film is annealed, reducing the donor defects decrease and improving the surface morphology of the ZnO films, leading to a decrease of response to the O_2_ gas. Notably, this phenomenon only exists in the first few measurements of the sensors. The response of the sensors stabilizes with the increase of the experimental period, which can be demonstrated by the XRD measurements of the ZnO films as shown in Figure 10. The ZnO film is characterized after 0, 1, 5 and 10 high-temperature experimental periods, respectively. It can be seen that the intensity of the ZnO (002) peak stops changing after 5 experimental periods, indicating the crystalline state of the film becomes stable.

### 3.4. Stability

**Figure 11:**
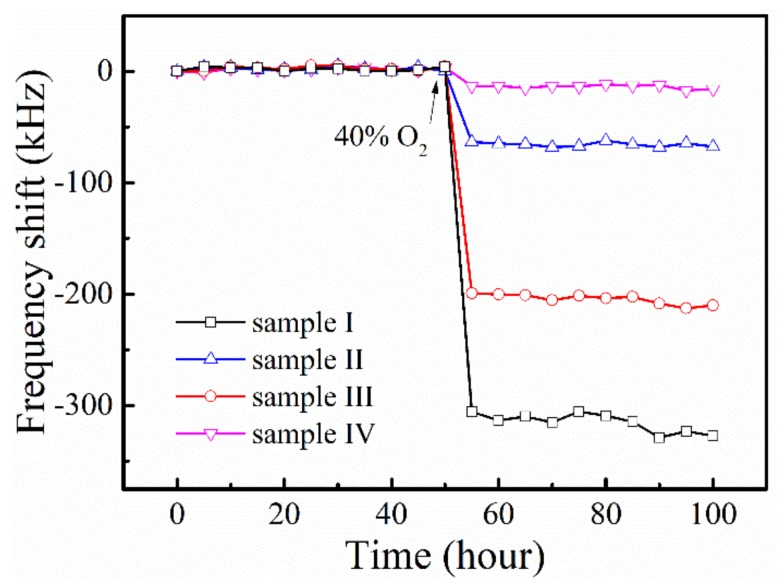
The measurement of stability of the sensors at operating temperature of 850 °C.

It is well known that the conducting electrodes and sensitive film would be damaged under long-term high-temperature measurement. In the previous work, the layered electrodes have proven to be reliable techniques to improve thermal stability of the SAW sensors. In this section, ZnO film-based SAW sensors are examined under high-temperature and oxidize rich conditions to investigate their stability. As shown in Figure 11, the sensors were tested for a period of 100 h at 850 °C. In the first half of the period, the sensors operated in the background gas environment, and then were exposed to the 40% concentration of O_2_ surroundings in the second half. The response data is recorded at every 5 h. The calculated standard error of the response of the sensors was less than 3%, indicating a good stability of the sensors. Notably, sample I possessed the largest standard error and the perturbation of the sensor enhanced when exposed to target gas. This is caused by the thermal annealing happening to the ZnO film in the long period the of high-temperature measurements process as discussed before [26]. Due to the additional link loss in the wireless detection method, measurements were only possible to 850 °C. However, the sensors still maintain good performance after the whole measurements process. In addition, with further measurements, no significant signal degradation of the sensors could be observed until now.

## 4. Conclusions

High-temperature oxygen sensors based on langasite substrate and ZnO-sensitive film were fabricated and tested up to 850 °C. ZnO films with different surface morphology were obtained by changing deposition conditions. The relationship and mechanisms between the micro structure of the ZnO film and gas-sensing response were systematically investigated. The behavior of the sensors was not only involved in the microstructure of the ZnO-sensitive films, but also affected by the operating temperature. On the one hand, the SAW sensors with coarse surface and large grain size of ZnO film exhibited a higher response than the others, as well as a longer response and recovery time. On the other hand, with increasing operating temperature, the frequency shift of the sensors increased. Meanwhile, the response and recovery time also increased slightly. It is important to note that no significant signal attenuation of the SAW sensors was detected when heated up to 850 °C in a long measurement term. However, the sensors almost reached maximum range when exposed to 40% concentration of O_2_. We speculate that this is owing to the small specific surface area of the ZnO film obtained by the PLD method. Hence, further research to enlarge the specific surface area by surface modification and element doping is underway. The experimental results show a good performance of the sensor, indicating huge potential use of the sensors in high-temperature sensing applications.

## Figures and Tables

**Table 1:** The parameters of the samples prepared in this work.

Sample Number	*T*_d_ (°C)	*t*_ZnO_ (nm)	*FWHM of ZnO Film (°)
I	25	204.5	10.2
II	200	202.6	6.4
III	400	195.8	3.7
IV	600	200.2	2.6

*FWHM: full width at half maximum
